# Ambient Air Pollution and Lipoprotein-Associated Phospholipase A_2_ in Survivors of Myocardial Infarction

**DOI:** 10.1289/ehp.1002681

**Published:** 2011-02-28

**Authors:** Irene Brüske, Regina Hampel, Zita Baumgärtner, Regina Rückerl, Sonja Greven, Wolfgang Koenig, Annette Peters, Alexandra Schneider

**Affiliations:** 1 Institute of Epidemiology II, Helmholtz Zentrum München, German Research Center for Environmental Health, Neuherberg, Germany; 2 Department of Epidemiology, Institut für Medizinische Informatik Biometrie und Epidemiologie, and; 3 Department of Statistics, Ludwig-Maximilians-Universität München, Munich, Germany; 4 Department of Cardiology, University of Ulm Medical Center, Ulm, Germany

**Keywords:** air pollution, atherosclerosis, epidemiology, inflammation, lipoprotein-associated phospholipase A_2_, myocardial infarction, panel study

## Abstract

Background: Increasing evidence suggests a proatherogenic role for lipoprotein-associated phospholipase A_2_ (Lp-PLA2). A meta-analysis of published cohorts has shown that Lp-PLA2 is an independent predictor of coronary heart disease events and stroke.

Objective: In this study, we investigated whether the association between air pollution and cardiovascular disease might be partly explained by increased Lp-PLA2 mass in response to exposure.

Methods: A prospective longitudinal study of 200 patients who had had a myocardial infarction was performed in Augsburg, Germany. Up to six repeated clinical examinations were scheduled every 4–6 weeks between May 2003 and March 2004. Supplementary to the multicenter AIRGENE protocol, we assessed repeated plasma Lp-PLA2 concentrations. Air pollution data from a fixed monitoring site representing urban background concentrations were collected. We measured hourly means of particle mass [particulate matter (PM) < 10 µm (PM_10_) and PM < 2.5 µm (PM_2.5_) in aerodynamic diameter] and particle number concentrations (PNCs), as well as the gaseous air pollutants carbon monoxide (CO), sulfur dioxide (SO_2_), ozone (O_3_), nitric oxide (NO), and nitrogen dioxide (NO_2_). Data were analyzed using mixed models with random patient effects.

Results: Lp-PLA2 showed a positive association with PM_10_, PM_2.5_, and PNCs, as well as with CO, NO_2_, NO, and SO_2_ 4–5 days before blood withdrawal (lag 4–5). A positive association with O_3_ was much more immediate (lag 0). However, inverse associations with some pollutants were evident at shorter time lags.

Conclusion: These preliminary findings should be replicated in other study populations because they suggest that the accumulation of acute and subacute effects or the chronic exposure to ambient particulate and gaseous air pollution may result in the promotion of atherosclerosis, mediated, at least in part, by increased levels of Lp-PLA2.

Most epidemiological studies have shown an elevated risk for cardiovascular events associated with exposure to fine particulate matter (PM) (de Hartog 2003; de Hartog et al. 2009; Peters et al. 2001, 2004; Schwartz 1997). PM has been associated with increased risks of myocardial infarction (MI), stroke, arrhythmia, and heart failure within hours to days of exposure in susceptible individuals. In addition, air pollutants have been linked with intermediate outcomes that may reflect mechanistic links, including endothelial dysfunction and vasoconstriction, increased blood pressure (BP), prothrombotic and coagulant changes, systemic inflammatory and oxidative stress responses, autonomic imbalance and arrhythmias, and the progression of atherosclerosis (Brook et al. 2010).

Lipoprotein-associated phospholipase A_2_ (Lp-PLA2), a member of the phospholipase superfamily, is an enzyme produced by monocytes and macrophages, T cells, and mast cells. Oxidized low-density lipoprotein (LDL) within the subendothelial space is converted by Lp-PLA2 into oxidized free fatty acids and lysophoshatidylcholine (Macphee et al. 1999; Zalewski and Macphee 2005). These products trigger an inflammatory cascade by stimulating the expression of adhesion molecules and the release of cytokines by endothelial cells and plaque-based macrophages, which recruit more monocytes into the subendothelial space where they become activated and differentiate into macrophages. The fact that Lp-PLA2 is produced locally within atherosclerotic lesions itself likely accounts for its high specificity for vascular as opposed to systematic inflammation (Lerman and McConnell 2008). Plasma Lp-PLA2 levels are not affected by systemic inflammatory diseases such as rheumatoid arthritis, osteoarthritis, or chronic obstructive pulmonary disease (Lerman and McConnell 2008).

Plasma Lp-PLA2 is bound mainly to LDL. Epidemiological studies have found consistent and statistically significant positive associations between Lp-PLA2 mass or activity and coronary artery disease and stroke (Anderson 2008; Gorelick 2008; Koenig and Khuseyinova 2009; Koenig et al. 2009). Lp-PLA2 is a marker of cardiovascular risk independent of and in addition to traditional risk factors [Lp-PLA(2) Studies Collaboration et al. 2010], and Lp-PLA2 may be directly involved in the causal pathway of plaque inflammation and the formation of rupture-prone plaques (Lerman and McConnell 2008).

This study was performed to investigate whether exposure to ambient particulate and gaseous air pollutants is associated with a systemic increase in Lp-PLA2 levels, consistent with our hypothesis that Lp-PLA2 may be a potential mediator in the pathway between air pollution and cardiovascular disease.

## Materials and Methods

*Study design and study population.* A prospective longitudinal study of post-MI patients was conducted in Augsburg, Germany, and two adjacent counties (a total of 300,000 inhabits were living in the three counties at the time of the study) as part of a study on Air Pollution and Inflammatory Response in Myocardial Infarction Survivors: Gene-Environment Interaction in a High Risk Group (AIRGENE) (Peters et al. 2007). Up to six repeated clinical examinations were scheduled every 4–6 weeks between May 2003 and March 2004 in six European cities (Athens, Greece; Augsburg, Germany; Barcelona, Spain; Helsinki, Finland; Rome, Italy; and Stockholm, Sweden). In total, 1,142 clinical examinations were performed. Supplementary to the multicenter AIRGENE protocol, we assessed repeated Lp-PLA2 plasma levels only in participants from the Augsburg region.

Candidates for the study were identified from the Augsburg MI registry. MI was defined according to recommendations by the European Society of Cardiology/American College of Cardiology Committee (Alpert et al. 2000). Eligible study participants were between 35 and 80 years of age and had had an MI > 3 months and up to 6 years prior to the start of the study, corresponding to an MI that occurred between 1997 and 2003. We excluded patients who had had an MI or interventional procedures (percutaneous transluminal coronary angioplasty, coronary artery bypass surgery) < 3 months before the beginning of the study and patients who had chronic inflammatory diseases or who were taking oral anticoagulant medications, which can potentially affect inflammatory and hemostatic markers. All study participants in Augsburg lived within a 10-km radius of the central monitoring station; participants who had left this area for > 1 month during the study period were also excluded. Only current nonsmokers or occasional smokers (< 1 cigarette/day) were recruited. Ex-smokers were considered nonsmokers if they had quit smoking at least 3 months before the start of the study. The study protocol was approved by the human subjects committee, Bayerische Landesärztekammer, and written informed consent was obtained from all patients at their first clinical visit.

*Clinical measurements.* At the first visit, a baseline questionnaire was administered regarding patient characteristics such as health status, history of coronary heart disease and other comorbidities, smoking history, and socioeconomic status. All medication taken during the course of the study was recorded, including brand name, dose, and intake pattern. A blood serum sample was drawn to determine serum lipids including total cholesterol and high-density lipoprotein (HDL) cholesterol, glycated hemoglobin (HbA1c), which is proportional to average blood glucose concentration over the previous 4 weeks to 3 months, as well as *N-*terminal proB-type natriuretic peptide (NT-proBNP), an indicator of heart failure.

In general, six repeated clinical visits were scheduled every 4–6 weeks on the same weekday and at the same time of the day to minimize the impact of weekly and circadian variation. If the participant was unable to comply with this criterion, another day of the week was selected with an appointment at the same hour (plus or minus 1 hr). If volunteers suffered from acute infections such as a cold or influenza up to 3 days before the clinical visit, examinations were postponed or the respective blood sample was excluded from analyses.

A 7-day recall on medication intake was obtained. Venous ethylenediamine tetraacetic acid (EDTA)-plasma samples were collected for the determination of Lp-PLA2 mass levels and C-reactive protein (CRP). Samples were cooled and stored at 4°C until further processing within a maximum of 4 hr after blood draw. EDTA-blood was centrifuged at 4°C in a precooled centrifuge for 20 min at 2,500 × *g*. Plasma aliquots were kept at –80°C until they were shipped on dry ice to the laboratory at the University of Ulm Medical Center in Ulm, Germany, for analyses. Plasma levels of Lp-PLA2 were determined with a commercially available Lp-PLA2-ELISA (PLAC test; diaDexus, Inc., San Francisco, CA, USA). CRP concentrations were measured by a high-sensitivity latex-enhanced immunonephelometry (Dade Behring GmbH, Marburg, Germany). The detection limits were 1.3 ng/mL for Lp-PLA2 and 0.16 mg/L for CRP. Duplicate blood samples were received from 11 patients and were blinded before determining Lp-PLA2 levels. The relative difference was 2.7% (–7.4 to 13.8) and the *R*-square (0.98) of the blinded duplicate blood samples showed a good agreement of the measurements. Further quality control procedures included 147 repeat measurements of samples from 104 participants three times each. The coefficient of variation was 2.07% (0–11.8%), and the pairwise Spearman correlation coefficients were 0.99 and 0.97 between the three Lp-PLA2 measurements.

*Air pollution and meteorological data.* We collected air pollution data from a fixed monitoring site that represented urban background concentrations according to standard procedures already employed in several European studies of air pollution (Aalto et al. 2005; Katsouyanni et al. 1996). Exposure assessment included hourly means of particulate matter (PM) < 10 µm (PM_10_), and PM < 2.5 µm (PM_2.5_) in aerodynamic diameter, and particle number concentrations (PNCs; < 100 nm), which represent ultrafine particles; the assessment also included gaseous air pollutants [carbon monoxide (CO), sulfur dioxide (SO_2_), ozone (O_3_), nitric oxide (NO), and nitrogen dioxide (NO_2_)] and meteorological variables (air temperature, relative humidity, barometric pressure). Meteorological data were obtained through the Deutscher Wetterdienst (Offenbach, Germany; air monitoring network and meteorological services). Daily average concentrations of particles and gases and the 8-hr average of O_3_ were calculated if at least 75% of the observations were available. PNC was measured using a condensation particle counter (model 3022A; TSI Inc., St. Paul, MN, USA).

Missing data on the aggregate level were replaced using a formula adapted from the Air Pollution and Health—A European Approach method (Katsouyanni et al. 1996; Rückerl et al. 2007) [for details, see Supplemental Material (doi:10.1289/ehp.1002681)]. If all measurements were missing for 1 day, the averages from the day before and the day after were taken. If data were missing for > 24 hr, data were not replaced.

*Statistical analyses.* For each person and visit, we calculated individual 24-hr average exposures immediately preceding the clinical visit (lag 0) and up to 5 days before the visit (lag 1–lag 5).

Data were analyzed using mixed models with random patient effects to account for the repeated measures data structure. To model correlations between the repeated measures in each patient, we assumed a compound symmetry structure for the covariance matrix, as the half-life of Lp-PLA2 is shorter than the intervals between visits. Penalized splines (P-splines) in the additive mixed-models framework were used to allow for nonparametric exposure–response functions (Greven et al. 2006).

We built a confounder model without including air pollutants. In the first step, time-invariant factors with potential impacts on average Lp-PLA2 concentrations were evaluated to permit the assumption of a normally distributed random patient intercept. These factors included age, sex, body mass index (BMI; kilograms per square meter), smoking, number of MIs, time since last MI, level of education, and working status that were classified at the time of enrollment. As further possible indicators for Lp-PLA2 levels, we considered self-reported overall health status, doctor’s diagnosis of diabetes, osteoarthritis, hay fever, chronic renal diseases, hypertension, respiratory diseases and symptoms, diagnosis of cardiovascular diseases (angina pectoris, arrhythmias, congestive heart failure, and stroke), the presence of a cardiac pacemaker, blood pressure, total and HDL cholesterol, NT-proBNP, and HbA1c levels.

In the second step, time-varying variables were added. To assure sufficient adjustment for season and meteorology, we forced long-term time trend and air temperature into the model. Additionally, relative humidity and barometric pressure, time of the day, and weekday were included if this adjustment proved necessary. P-splines were used to model continuous covariables and were compared with linear terms and polynomials of degree 2 and 3. All decisions on goodness-of-fit were based on Akaike Information Criterion (AIC; 1973).

After completing the confounder model selection [see Supplemental Material, Table 1 (doi:10.1289/ehp.1002681)], we added single air pollution lags, and the effects were estimated linearly. The exposure–response function for the air pollutants was assessed using P-splines and showed no consistent deviation from linearity across lags, so only estimates for the linear function are presented. Effect estimates are presented as percent change of the mean of Lp-PLA2 levels together with 95% confidence intervals (CIs) based on an increase in air pollution concentrations from the first to the third quartile [interquartile range (IQR): difference between the third and first quartile]. Data were analyzed using SAS (version 9.1; SAS Institute Inc., Cary, NC, USA).

**Table 1:** Characteristics of the study population: 200 MI survivors in Augsburg, Germany, 14 May 2003–24 February 2004.

Table 1. Characteristics of the study population: 200 MI survivors in Augsburg, Germany, 14 May 2003–24 February 2004.	
Characteristic	Mean ± SD or total *n* (%)
Age (years)	61.9 ± 9.0
Last MI to study (years)	2.1 ± 0.9
BMI (kg/m²)	28.8 ± 4.0
Alcohol intake per day (g)	18.5 ± 22.6
Systolic blood pressure (mmHg)	128.4 ± 19.9
Total cholesterol (mg/dL)	181.0 ± 38.8
HDL (mg/dL)	47.9 ± 11.8
HbA1c (%)	5.6 ± 0.7
CRP (mg/L)	2.2 ± 4.5
Lp-PLA2 (ng/mL)	188.6 ± 51.1
Sex (male)	164 (82)
Smoking	
Ex-smokers	126 (63)
Occasional smokers	9 (4.5)
Never smokers	65 (32.5)
No. of MIs	
1	175 (88)
> 1	25 (12)
BMI (kg/m²)	
≤ 25	31 (16)
> 25	168 (84)
HbA1c (%)	
< 6.5	181 (90)
≥ 6.5	19 (10)
Medical history	
Angina pectoris	42 (21)
Arrhythmias	48 (24)
Congestive heart failure	26 (13)
Stroke	13 (7)
Diabetes mellitus	35 (18)
Hypertension	102 (51)
Chronic bronchitis	16 (8)
Asthma	9 (5)
Emphysema	2 (1)
Hay fever	20 (10)
Chronic renal disease	10 (5)
Osteoarthritis	35 (18)
Medications	
Beta blockers	183 (92)
ACE inhibitors	139 (70)
Diuretics	79 (40)
Statins	173 (87)
Fibrates	2 (1)
ACE, angiotensin converting enzyme.	

*Effect modification.* Binary interaction variables were added to the model to estimate the air pollution effects of the corresponding subgroups. Interaction variables were sex (male vs. female), season (April–September vs. October–March), BMI (≤ 25 kg/m² vs. > 25 kg/m²), and CRP (≤ 3 mg/L vs. > 3 mg/L). An effect modification was assumed to be present based on *p*-value < 0.05.

*Sensitivity analyses.* To check the robustness of our models, we performed several sensitivity analyses. Air pollution effects were also estimated by adjusting for the time-varying confounders only. In a further sensitivity analysis, models were additionally adjusted for the intake of antiinflammatory and antirheumatic medication as well as diuretics and drugs for chronic obstructive pulmonary disease up to 7 days before blood withdrawal.

We also estimated the association of each air pollutant with Lp-PLA2 level using an unconstrained distributed lag model that included air pollution levels for the same day and the 5 previous days simultaneously.

## Results

*Study population.* Baseline characteristics of the study population, as well as laboratory results, medical history, physical activity, and medication, are shown in Table 1. In total, 200 MI survivors were recruited who fulfilled the inclusion criteria and had at least two valid blood samples taken. Study participants were mostly men and consisted of never smokers (32.5%), occasional smokers (4.5%), and ex-smokers (63%). On average, they were 62 years old and had 5.7 repeat visits to the study center. The total number of analyzed blood samples was 1,142. CRP levels were in the range of ≤ 3 mg/L at 82% (*n* = 932) of blood samples.

*Lp-PLA2 data.* Lp-PLA2 was nearly normally distributed with a mean ± SD level of 189 ± 51 ng/mL. The mean level was higher in men (190 ± 52 ng/mL) than in women (181 ± 44 ng/mL), and it was higher in winter (191 ± 54 ng/mL) than in summer (180 ± 49 ng/mL). For 69% of all visits, Lp-PLA2 measurements were higher in winter. Some participants showed consistently high or low Lp-PLA2-levels during the whole study period.

*Air pollutants.* The distributions of the 24-hr average concentrations of the particulate and gaseous pollutants as well as meteorological data are given in Table 2. Measurement values characterize the population average exposures typical for urban background pollution mainly due to traffic emissions and domestic heating during the winter season.

**Table 2:** Description of the 24-hr average concentrations of the particulate and gaseous pollutants as well as meteorological data in Augsburg, Germany, 14 May 2003–24 February 2004.

Table 2. Description of the 24-hr average concentrations of the particulate and gaseous pollutants as well as meteorological data in Augsburg, Germany, 14 May 2003–24 February 2004.									
Pollutant	*n*	Sites	Mean ± SD	Minimum	25%	Median	75%	Maximum	IQR
PNC (1/cm³)	198	1	11,876 ± 6,077	2,764	7,085	10,824	14,440	32,528	7,355
PM_2.5_ (μg/m³)	283	1	17.4 ± 6.2	6.2	12.2	16.9	21.2	38.7	8.9
PM_10_ (μg/m³)	286	3	33.1 ± 13.4	7.1	22.0	32.7	42.7	70.9	20.6
CO (mg/m³)	287	2	0.6 ± 0.2	0.3	0.4	0.5	0.7	1.7	0.2
NO_2_ (μg/m³)	287	3	40.0 ± 10.9	13.3	32.7	39.2	46.5	71.9	13.8
NO (μg/m³)	287	3	30.0 ± 24.0	7.6	17.2	23.0	32.3	176.4	15.1
SO_2_ (μg/m³)	287	2	3.0 ± 1.3	2.0	2.1	2.5	3.3	8.9	1.2
O_3_ (8-hr average) (μg/m³)	286	1	54.4 ± 36.0	3.0	20.4	56.0	82.3	137.9	61.9
Air temperature (°C)	285	1	10.2 ± 9.6	–9.4	1.9	10.3	19.6	27.6	17.7
Relative humidity (%)	285	1	69.0 ± 14.2	38.1	80.2	68.9	80.2	94.3	23.5
Barometric pressure (hPa)	285	1	1018.7 ± 6.9	991.0	1023.6	1019.8	1023.6	1032.3	8.4

Using Spearman correlation coefficient, we found that PNC highly correlated with CO (micrograms per cubic meter; *r* = 0.74), NO (micrograms per cubic meter; *r* = 0.74), and SO_2_ (micrograms per cubic meter; *r* = 0.77) (Table 3). NO and CO showed the highest correlation (*r* = 0.82). PM_10_ and PM_2.5_ were highly correlated (*r* = 0.93), whereas UFP was only moderately correlated with PM_10_ (*r* = 0.41) and PM_2.5_ (*r* = 0.35). A low correlation was also seen for NO_2_ with PNC (*r* = 0.54), PM_2.5_ (*r* = 0.57), PM_10_ (*r* = 0.66), and CO (*r* = 0.67). O_3_ concentrations were negatively correlated with all pollutants and with relative humidity (*r* = –0.74) and positively correlated with temperature (*r* = 0.80).

**Table 3:** Spearman correlation coefficients of the 24-hr average concentrations of the particulate and gaseous pollutants as well as meteorological data in Augsburg, Germany, 14 May 2003–24 February 2004.

Table 3. Spearman correlation coefficients of the 24-hr average concentrations of the particulate and gaseous pollutants as well as meteorological data in Augsburg, Germany, 14 May 2003–24 February 2004.																				
Pollutant		PNC		PM_2.5_		PM_10_		CO		NO_2_		NO		SO_2_		O_3_		Temperature		Relative humidity
PNC		1																		
PM_2.5_		0.35		1																
PM_10_		0.41		0.93		1														
CO		0.74		0.58		0.61		1												
NO_2_		0.54		0.57		0.66		0.67		1										
NO		0.74		0.25		0.35		0.82		0.54		1								
SO_2_		0.77		0.42		0.43		0.63		0.51		0.60		1						
O_3_ (8-hr average)		–0.67		–0.08		–0.10		–0.66		–0.11		–0.73		–0.45		1				
Air temperature		–0.61		0.12		0.12		–0.42		0.10		–0.50		–0.38		0.80		1		
Relative humidity		0.23		–0.12		–0.14		0.34		–0.22		0.44		0.11		–0.74		–0.73		1
Barometric pressure		0.06		0.11		0.20		0.05		0.20		0.05		0.06		0.07		0.13		–0.29

Particulate and gaseous air pollutants during the study period are shown in Supplemental Material, Figure 1 (doi:10.1289/ehp.1002681).

**Figure 1:**
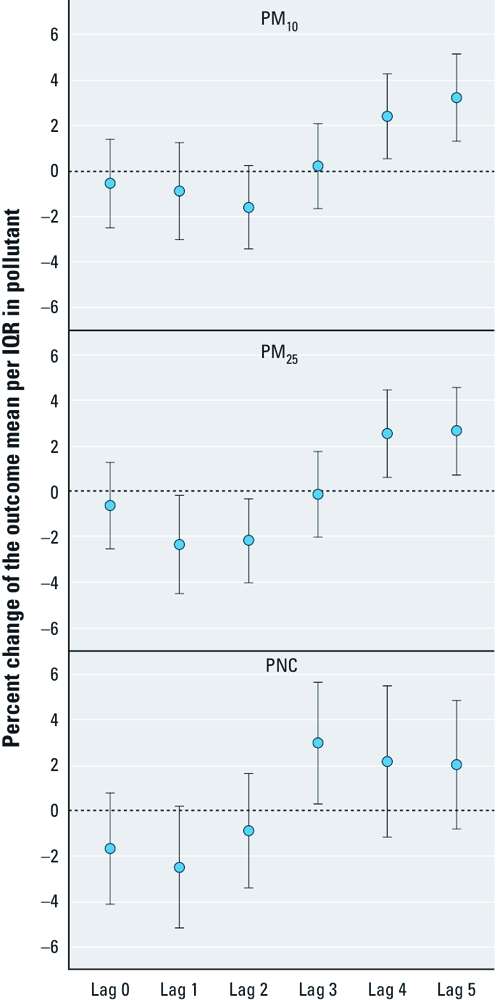
Percent changes of the mean Lp-PLA2 per IQR (difference between the third and first quartile) in particulate air pollutants together with 95% CIs in Augsburg, Germany, 14 May 2003–24 February 2004.

*Regression results.* Based on AIC, we selected seven time-invariant factors of 46 variables for the final confounder model: systolic blood pressure, serum cholesterol, time between MI and beginning of the study, number of MIs (0, 1, > 1), congestive heart failure (yes/no), alcohol intake (grams per day), and HbA1c (percent) < 6.5 (yes/no). In addition, the following time-varying factors were included in the final confounder model: trend (P-splines), weekday, 48-hr average of air temperature (P-splines) of the same and the previous day, 24-hr average relative humidity (P-splines) 4 days before the blood withdrawal, and barometric pressure of the same day (P-splines).

**Figure 2:**
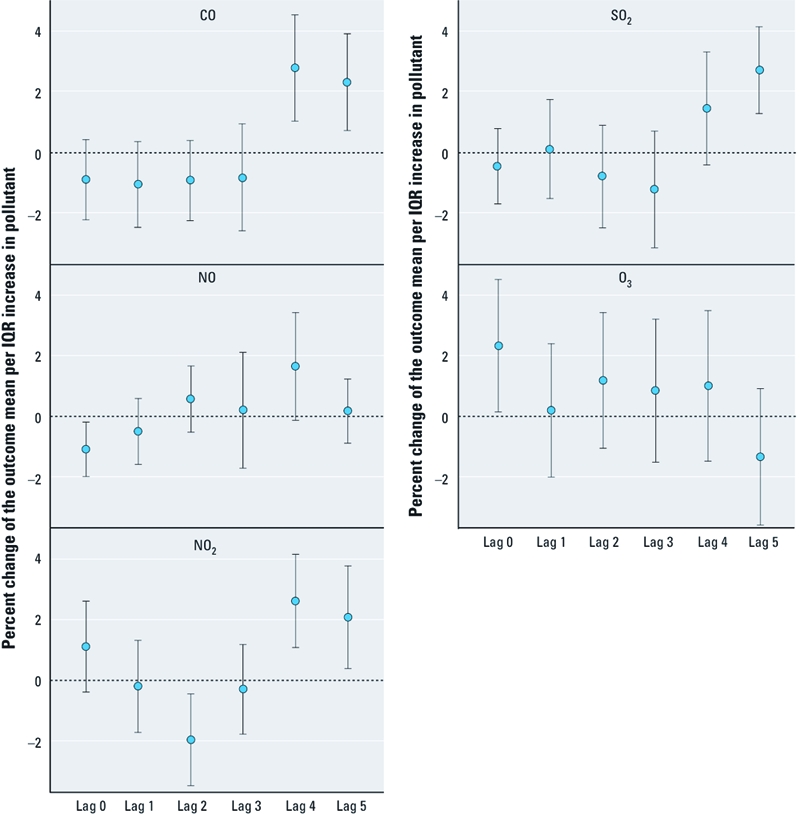
Percent changes of the mean Lp-PLA2 per IQR (difference between the third and first quartile) in gaseous air pollutants together with 95% CIs in Augsburg, Germany, 14 May 2003 to 24 February 2004.

IQR increases in PM_10_ and PM_2.5_ were associated with a maximum percent change in plasma Lp-PLA2 of 3.23 (95% CI, 1.31–5.16) and 2.65 (95% CI, 0.75–4.56) at lag 5, respectively, and an IQR increase in PNC was associated with a maximum percent change of 2.97 (95% CI, 0.31–5.64) at lag 3 (Figure 1). For IQR increases in gaseous pollutants, the maximum percent change in Lp-PLA2 was observed 4 days before blood withdrawal: 2.79 (95% CI, 1.04–4.54) for CO; 2.62 (95% CI, 1.08–4.16) for NO_2_; and 1.66 (95% CI, –0.13 to 3.45) for NO (Figure 2). The maximum percent change in Lp-PLA2 with an IQR increase in SO_2_ was observed at lag 5: 2.73 (95% CI, 1.30–4.17). However, IQR increases in PM_10_, PM_2.5_, PNC, and NO_2_ were inversely associated with Lp-PLA2 levels at lag 1 and lag 2. The association between O_3_ and Lp-PLA2 followed a completely different time pattern, with a maximum percent change of 2.34 (95% CI, 0.15–4.54) at lag 0.

*Effect modification.* Associations between air pollutants and Lp-PLA2 were not modified by sex or CRP level (*p*-value of interaction > 0.05; data not shown). Overall, associations of PM_10_, PM_2.5_, and NO_2_ with Lp-PLA2 tended to be stronger in normal weight (BMI ≤ 25 kg/m²) participants than in overweight participants [Supplemental Material, Figure 2, left half (doi:10.1289/ehp.1002681)]. Season was a strong effect modifier [Supplemental Material, Figure 2, right half (doi:10.1289/ehp.1002681)]. The association of Lp-PLA2 with PM_10_ and PM_2.5_, as well as with NO_2_, NO, and CO for lag 3-4 was seen in winter, but not in summer. For *p*-values of interaction, refer to Figure 2 in Supplemental Material (doi:10.1289/ehp.1002681).

*Sensitivity analyses.* Excluding three occasional smokers with 17 Lp-PLA2 measurements in a sensitivity analysis had no effect on the results.

Adjusting for time-invariant confounders only or for time-invariant factors and medications only did not lead to statistically significant changes in the air pollution Lp-PLA2 response. The lag pattern of air pollution associations did not change when using distributed lag models that accounted for all lag periods simultaneously. However, the associations tended to be less pronounced.

## Discussion

To our knowledge, this panel study of MI survivors is the first human study to examine the relation between air pollution and plasma Lp-PLA2 mass levels. Inverse associations were observed for particulate exposure and NO_2_ with Lp-PLA2 at lag days 1–2, and positive associations were estimated for all particulate and gaseous pollutants except O_3_ with Lp-PLA2 lagged 4 and 5 days. Our finding of a positive association between all air pollutants and Lp-PLA2 levels to lag 4–lag 5 was observed in the single pollutant model as well as in the distributed lag model. Information on the physiological behavior of Lp-PLA2 levels is not sufficient to determine whether the early (1–2 days) inverse associations and later positive associations (4–5 days) observed between air pollutants and Lp-PLA2 levels in our study population are biologically plausible. The immediate positive association with O_3_ suggests a different physiological mechanism, if associations represent causal effects. The independent effects of the various pollutants could not be estimated because of the interrelationship among most pollutants. Effects of PM air pollution on LDL levels could potentially confound associations between air pollutants and Lp-PLA2, but adjusting for LDL at baseline examination had practically no effect on associations.

As part of the AIRGENE study, repeated measurements of markers of inflammation (interleukin 6, fibrinogen, and CRP) were compared with concurrent levels of air pollution. An immediate association (12–17 hr) of interleukin 6 was seen, especially in relation to PNC, but no consistent associations were found for CRP, which was attributed to a widespread intake of statins in the study population of MI survivors (Rückerl et al. 2007). Based on a subset of the same study population, we found that associations between air pollution and Lp-PLA2 levels occurred later than associations with inflammatory markers. Furthermore, individual mean levels of Lp-PLA2 did not correlate with individual mean levels of CRP (*r* = 0.03), fibrinogen (*r* = –0.08), or interleukin-6 (*r* = –0.04).

We have no explanation for why the association of Lp-PLA2 with PM_10_ and PM_2.5_, as well as with NO_2_, NO, and CO for lag 3–lag 4, was seen only in winter, but not in summer. It might be related to additional air pollutants in winter not measured in the study or to different human metabolism in winter; however, these explanations are highly speculative. Moreover, we cannot explain why the increase of Lp-PLA2 tended to be stronger in normal weight (BMI ≤ 25 kg /m²) compared with overweighed study participants.

Based on their *in vitro* study, Beck-Speier et al. (2005) proposed that effects of particles on alveolar macrophage cell membranes or cell membrane receptors may trigger the extracellular signal-regulated kinase 1,2 (ERK 1,2) cascade via mitogen-activated protein kinase kinase1, resulting in activation of cytosolic and secretory Lp-PLA2. Both Lp-PLA2 enzymes hydrolyze correspondingly substituted phospholipids to produce arachidonic acid. Arachidonic acid, released by cytosolic PLA2, may stimulate secretory phospholipids A2 to amplify its liberation, as proposed by Balsinde et al. (1998).

In a study of the biological variability of Lp-PLA2 (Lerman and McConnell 2008), the percent coefficient of variation of Lp-PLA2 levels within an individual was rather low (10%), similar to LDL cholesterol. This finding was confirmed recently in our AIRGENE study population (Khuseyinova et al. 2008) in which the within-subject variation in plasma Lp-PLA2 concentration was substantially smaller than the between-subject variation. For this reason, Lp-PLA2 can be reliably followed serially over time rendering it a potentially useful clinical risk marker of CVD. Due to this stability, one would expect only small changes associated with air pollution. However, because we did not evaluate the within-individual changes in Lp-PLA1 concentrations, the clinical relevance of our observations remains uncertain.

*Strengths and limitations.* Our study offers an exploratory post-hoc analysis of the impact of exposure to ambient particulate and gaseous air pollutants on Lp-PLA2 levels. These data were collected in the German subset of the main AIRGENE study population and could be analyzed only with comparatively low power. The observed associations should be regarded as preliminary.

The air pollution measurements were made at a single monitoring site, which may not adequately reflect individual exposure, especially for PNC (Puustinen et al. 2007). Assuming a nondifferential misclassification, the true effect of PNC on Lp-PLA2 may have been underestimated. Cyrys et al. (2008) investigated the temporal and spatial variation of PNC at four urban background monitoring sites in Augsburg, Germany. The authors observed a high temporal correlation of PNC across the city area of Augsburg. This finding suggests that in epidemiological time-series studies, the use of one single ambient monitoring site is an adequate approach for characterizing exposure to ultrafine particles.

The study was designed to assess the impact of ambient air pollution on Lp-PLA2 levels in a cohort of MI survivors assuming a specific susceptibility. The study population was prescribed evidence-based cardiovascular medication for the post-MI period. Medication is an important effect modifier in this study that we could not take into account adequately. HMG-CoA reductase inhibitors (statins), for example, can reduce Lp-PLA2 concentrations in plasma by approximately 15–20% (McConnell and Hoefner 2006), but the effect of the medication could not be examined in detail because nearly all patients (87%) took statins. Other medications (e.g., angiotensin II receptor antagonists, ezetimibe, fish oil, and niacin) (Hill et al. 2009) may also have altered LpPLA2 levels. Potential intermittent adherence to medication may have biased the observed association. For subgroup analyses of different medication groups, the sample size, and thus the power of the study, was too small.

Furthermore, the study population was 86% male with only 36 female patients and also was restricted with regard to age, disease, and medication status. Therefore, findings may not be generalizable to the general population.

All blood samples were collected in the fasting state under standardized conditions and mostly at the same time (98%) of the same weekday (95%) to minimize the impact of circadian and day-to-day variation. The Lp-PLA2 mass, but not the activity, was measured. Although previous studies have shown a correlation (*r* ~ 0.6) between activity and mass (Iribarren et al. 2005; Koenig et al. 2006), this correlation may be weakened if an individual has a polymorphism or mutation that influences activity of the gene product (McConnell and Hoefner 2006).

## Conclusion

The daily time-series analysis showed a positive association of Lp-PLA2 mass levels with PM_10_, PM_2.5_, and PNC, as well as CO, NO_2_, NO, and SO_2_ with a delay of 4–5 days, but also indicated inverse associations with some pollutants over shorter time lags. The accumulation of acute and subacute effects or the chronic exposure to ambient particulate and gaseous air pollution may result in the promotion of atherosclerosis, mediated at least in part by increased levels of Lp-PLA2 and subsequently increased levels of their major pro-atherogenic and pro-inflammatory downstream products. However, these associations have not been evaluated previously, and results must be replicated before chance associations can be ruled out.

## Appendix 1.

The German AIRGENE study group comprises the following partners:

Helmholtz Zentrum München, German Research Center for Environment and Health, Institute of Epidemiology (Neuherberg, Germany): A. Peters (PI), I. Brüske, H. Chavez, J. Cyrys, U. Geruschkat, H. Grallert, S. Greven, A. Ibald-Mulli, T. Illig, H. Kirchmair, S. von Klot, M. Kolz, M. Marowsky-Koeppl, M. Mueller, R. Rückerl, A. Schaffrath Rosario, A. Schneider, H.-E. Wichmann.

Helmholtz Zentrum München German Research Center for Environmental Health, Institute of Health Economics and Health Care Management (Neuherberg, Germany): R. Holle, H. Nagl.

Helmholtz Zentrum München German Research Center for Environmental Health, KORA Study Center (Augsburg, Germany): I. Fabricius, C. Greschik, F. Günther, M. Haensel, U. Hahn, U. Kuch, C. Meisinger, M. Pietsch, E. Rempfer, G. Schaich, I. Schwarzwälder, B. Zeitler.

KORA Myocardial Infarction Registry (Augsburg, Germany): C. Meisinger.

University of Ulm, Medical Center, Department of Internal Medicine II – Cardiology (Ulm, Germany): W. Koenig, N. Khuseyinova, G. Trischler.

## Supplemental Material

(124 KB) PDF
