# Supplementary material for: Ambient Air Pollution and Lipoprotein-Associated Phospholipase A2 in Survivors of Myocardial Infarction
Source: Environ Health Perspect. 2011 Feb 28;119(7):921–6. doi: 10.1289/ehp.1002681 (PMC3223011; doi:10.1289/ehp.1002681)

Supplemental Material

Ambient Air Pollution and Lipoprotein Associated Phospholipase A_2 in Myocardial Infarction Survivors

Irene Brüske, Regina Hampel, Zita Baumgärtner, Regina Rückerl, Sonja Greven, Wolfgang Koenig, Annette Peters, Alexandra Schneider

Table 1: Confounders selected for the model

	Shape	***Lag***
Systolic blood pressure [mmHg]	*Linear*	
Serum cholesterol [mg/dl]	*Linear*	
Alcohol intake per day [g/day]	*Linear*	
Time Interval between last MI and start of study [years]	*Linear*	
Myocardial infarction (no vs. 1 vs. >1)	*Categorial*	
Hba1c (<6.5 vs. ≥6.5)	*Categorial*	
Heart failure (yes vs. no)	*Categorial*	
Long-term time trend	*P-Spline*	
Air temperature [°C]	*P-Spline*	*2-d average*
Relative humidity [%]	*P-Spline*	*24h-average lag 4*
Barometric pressure [kPa]	*P-Spline*	*24h-average lag 0*

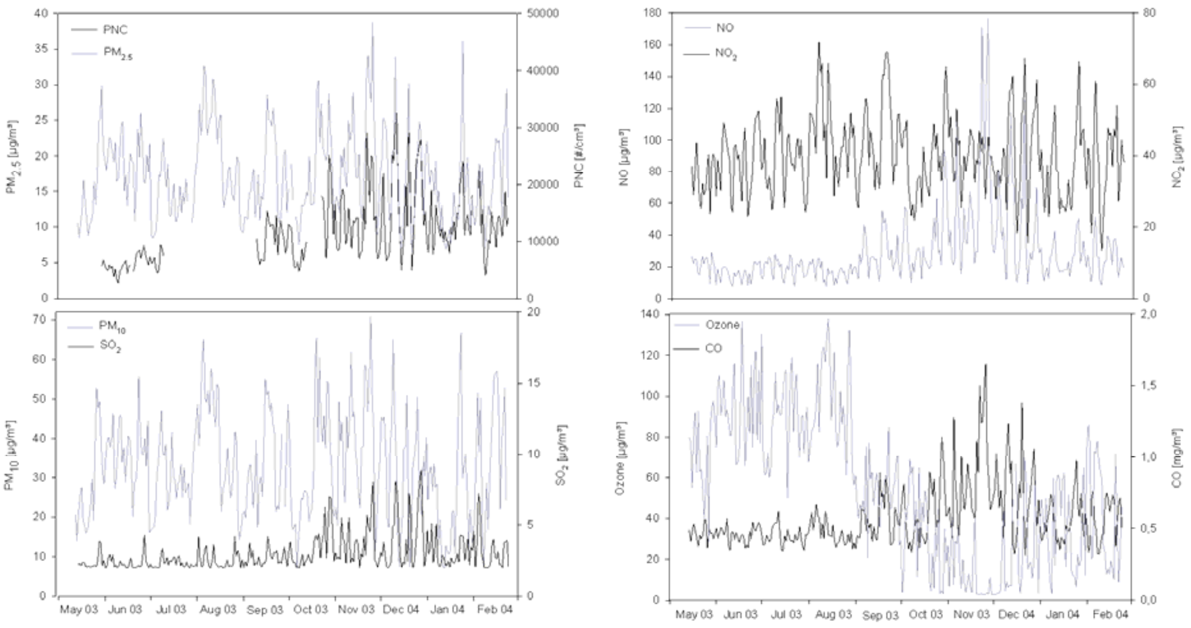

Figure 1 Particulate (PM_{10}, $PM_{2.5,}$ PNC) and gaseous (SO_2, No, NO_2, CO, Ozone) air pollution in Augsburg, Germany, May 14th 2003 to February 24th 2004

Figure 2: PM_{10} and NO_2 effects on Lp-PLA2 modified by BMI (<=25kg/m² vs. >25kg/m²) and season (Oct-Mar vs. Apr-Sep). P-values of interaction are indicated.

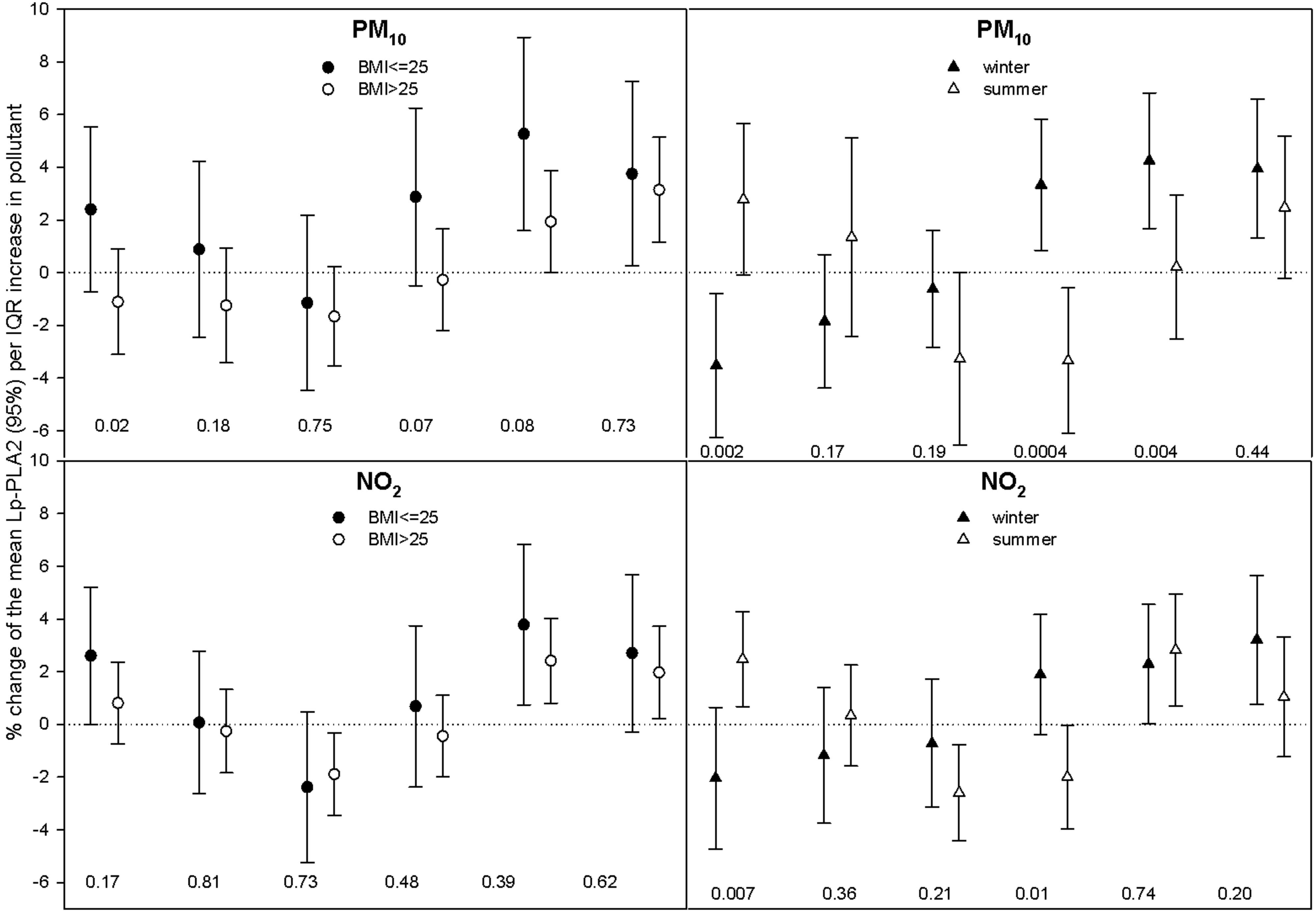

Supplement: (124 KB) PDF [file ehp.1002681.s001.pdf]
